# Metabolic Potential of *Epichloë* Endophytes for Host Grass Fungal Disease Resistance

**DOI:** 10.3390/microorganisms10010064

**Published:** 2021-12-29

**Authors:** Krishni Fernando, Priyanka Reddy, German C. Spangenberg, Simone J. Rochfort, Kathryn M. Guthridge

**Affiliations:** 1 Agriculture Victoria, AgriBio, Centre for AgriBioscience, Bundoora, VIC 3083, Australia; krishni.fernando@agriculture.vic.gov.au (K.F.); priyanka.reddy@agriculture.vic.gov.au (P.R.); german.spangenberg@agriculture.vic.gov.au (G.C.S.); simone.rochfort@agriculture.vic.gov.au (S.J.R.); 2 School of Applied Systems Biology, La Trobe University, Bundoora, VIC 3083, Australia

**Keywords:** antifungal metabolites, fungitoxic, pasture and turf protection, metabolite annotation, bioprospecting endophytes

## Abstract

Asexual species of the genus *Epichloë* (Clavicipitaceae, Ascomycota) form endosymbiotic associations with Pooidae grasses. This association is important both ecologically and to the pasture and turf industries, as the endophytic fungi confer a multitude of benefits to their host plant that improve competitive ability and performance such as growth promotion, abiotic stress tolerance, pest deterrence and increased host disease resistance. Biotic stress tolerance conferred by the production of bioprotective metabolites has a critical role in an industry context. While the known antimammalian and insecticidal toxins are well characterized due to their impact on livestock welfare, antimicrobial metabolites are less studied. Both pasture and turf grasses are challenged by many phytopathogenic diseases that result in significant economic losses and impact livestock health. Further investigations of *Epichloë* endophytes as natural biocontrol agents can be conducted on strains that are safe for animals. With the additional benefits of possessing host disease resistance, these strains would increase their commercial importance. Field reports have indicated that pasture grasses associated with *Epichloë* endophytes are superior in resisting fungal pathogens. However, only a few antifungal compounds have been identified and chemically characterized, and these from sexual (pathogenic) *Epichloë* species, rather than those utilized to enhance performance in turf and pasture industries. This review provides insight into the various strategies reported in identifying antifungal activity from *Epichloë* endophytes and, where described, the associated antifungal metabolites responsible for the activity.

## 1. Introduction

Pasture is one of the main food sources for livestock throughout the world. While pure pasture-based farming systems rely solely on pasture, mixed farming systems supplement with commodities such as cereals and grains. More consumers are shifting towards “grass-fed” livestock produce, as it caters for both increasing global food demands and social concerns for animal-welfare [1,2]. Novel solutions to sustainably manage food supply systems also address increasing demands for nutritious food [3,4]. There is a wide variety of pasture species used in agriculture including annual grasses, perennial grasses, legumes, and herbs. Forage grasses that are adaptable to a wide range of climatic conditions are extensively used in farming systems [5,6].

With changing climates and evolving pest and pathogen pressures, pasture and turf grasses are threatened by abiotic and biotic stresses with increasing severity [5,7,8,9]. It has long been known that *Epichloë* fungi of the family Clavicipitaceae, associated with Pooidae grasses such as *Lolium* spp. (e.g., perennial ryegrass, short-term ryegrass and tall fescue), improve host plant abiotic and biotic stress tolerance by producing bioactive metabolites [9,10,11,12]. Though *Epichloë* endophytes are historically well-characterised for their antimammalian and insecticidal alkaloid toxins, recent studies confirm that their bioactivities are not limited to these compounds alone [11,13,14,15]. Therefore, there is a requirement for an increase in applied research into *Epichloë* endophyte-derived bioactive metabolites to improve host grass performance and stress tolerance. This review focuses on prospects for *Epichloë* endophytes providing host plant disease resistance, and discusses modern experimental data analysis techniques for bioprospecting of antifungal metabolites in novel *Epichloë* strains.

## 2. *Epichloë* Endophytes

*Epichloë* fungi infecting grasses are categorized as sexual or asexual species based on their transmission mechanism. Sexually transmitting *Epichloë* spp. are known to cause choke disease in grasses during flowering, leading to reduced seed yield and aesthetic value of turf grasses [16,17]. In this process the fungus forms a stroma with spores and horizontal transmission occurs [16,17]. As they cause disease, sexual *Epichloë* spp. are considered pathogenic even though they live asymptomatically in the host plant during vegetative growth stages. Asexual *Epichloë* spp. are clonal, transmit vertically by infecting the seeds, and do not cause diseases to the host grass in any growth stage of the host plant [16]. Therefore, as they provide significant performance benefits, the pasture and turf grass industries utilize asexual (endophytic) strains of *Epichloë* species.

Asexual *Epichloë* spp. form endophytic associations with cool-season grasses by colonizing leaves, pseudostems, seeds and seedlings. In this mutualistic relationship, the endophyte relies on the plant for vertical dissemination while also gaining shelter and nutrients throughout its life [13]. The production of functional metabolites triggered by the association greatly benefits the host plant by conferring abiotic and biotic stress tolerances such as improved seedling vigour, persistence, and enhanced growth [13,18]. While most of these benefits are advantageous in an agricultural scenario, *Epichloë* endophytes are also well known for producing toxic antimammalian alkaloids (lolitrem B—ryegrass staggers, ergovaline—fescue toxicosis). These alkaloid toxins are extremely harmful to grazing animals and cause significant economic losses to the livestock industry [19,20,21]. Consequently, studies related to their biological and chemical properties, genetics, biosynthetic pathway and modes of action have been the focus from as early as 1980s. The biosynthetic intermediaries of these compounds have also been characterized for biological activity and many have been selected as candidates for insecticides [22]. Previous reports on *Epichloë* endophytes have demonstrated that the alkaloids lolitrem B, ergovaline, *n*-acetylloline, *n*-formylloline and peramine possess insecticidal and invertebrate pest deterrence properties [20,23,24]. However, the broad chemical diversity and metabolic capacity of *Epichloë* endophytes relating to antimicrobial activity remain largely unknown.

Field, glasshouse and lab-based studies have established that *Epichloë* endophytes improve host plant disease resistance, and a few studies have identified the production of metabolites with novel antimicrobial properties that may play a role in host plant protection from phytopathogens [11,12,25]. However, in most of these studies wild-type strains such as SE (Standard Endophyte; also referred to as wild type or common endophyte, CE) or Ky31 (Kentucky31) were investigated rather than the animal friendly strains utilized in pastoral agriculture. Thus, to better exploit endophyte-mediated disease resistance, improve pasture and turf quality, and reduce the impact of phytopathogen disease on animal welfare, *Epichloë* strains that are safe for animals, should be investigated.

## 3. Novel Endophytes

The search for endophytes that do not produce metabolites toxic to mammalian grazers led to the discovery of novel endophytes and their use commercially, as shown by the many novel strains for which Plant Breeders’ Rights has been granted worldwide, and those marketed with registered trademarks (Table 1) [26,27,28]. The significant economic benefits of animal-safe endophyte strains that also improve pasture persistence by reducing the impact of invertebrate pests underpinned the investigation and exploration of high-performing endophyte infected pasture grasses [29,30,31]. Novel endophytes are typically screened and selected based on alkaloid profiles in planta, in particular low/no lolitrem B and low/no ergovaline combined with bioprotective (insect deterring) lolines and/or peramine. However, more recently, screening methods have been extended to biosynthetic pathways and gene clusters associated with these known alkaloids [27,29,32,33].

The search for animal-safe endophyte strains is important to the agricultural industry, with new endophytes being developed continuously (Table 1). Therefore, high-throughput methods and commercial standards are used regularly to detect the presence of alkaloids in pasture [34]. Those compounds that are not commercially available can also be isolated and purified using published methodologies [19,35]. The screening methods are, however, limited to the “known known” alkaloids—the well described *Epichloë*-derived compounds referred to in Table 1. Of the novel endophytes described on Table 1 only two, Nea 12 and Nea 23, have been investigated for production of antifungal metabolites in vitro and in planta [36,37]. Thus, further research is required to investigate the ‘known unknown’ metabolites, such as those responsible for disease resistance, that are beneficial to the pasture and turf grass related industries [38].

## 4. Disease Stress to Pasture Grasses

Pasture grasses are threatened by many pathogenic diseases, causing devastating losses to pasture yield and quality. These diseases include rusts, leaf spot diseases, blights, blotches, moulds, and wilts caused by pathogenic fungi, bacteria or viruses (Table 2).

In perennial ryegrass, crown rust (*Puccinia coronata*) is one of the most severe fungal disease-causing pathogens that affects foliage, causing substantial losses in pasture and turf grass industries. Severe rust infections can cause up to 37% loss in dry matter (DM) when infected plants are harvested and dried, and a 94% loss in fresh matter (FM) yield [39,40,41,42]. Water soluble carbohydrate content in rust-infected grasses is significantly lower compared to uninfected grasses, which in turn affects digestibility and palatability and can lead to low milk yields if used as feed [43,44,45]. Root growth is also reduced due to depleted carbohydrate reserves [46]. Further, the resultant increase in dead herbage tissue makes grass susceptible to *Pithomyces chartarum*, which causes serious disease of facial eczema in cattle and sheep [46,47].

Other foliage pathogens include *Drechslera sissans*, which causes significant production losses; disease incidence is significantly increased with high nitrogen (N) use conditions [44,48,49]. Though not prominent in Australia, snow mould (*Microdochium nivale*) and grey leaf spot (*Pyricularia grisea*) are another two serious diseases causing yield losses in perennial ryegrass and tall fescue in the northern hemisphere [50,51].

As well as the effect on foliage, several fungal pathogens also directly infect the inflorescence, thus altering seed yield. The most significant of these are stem rust (*Puccinia graminis*) and blind seed disease (*Gloeotinia granigena*), which both occur on seed heads on a wide range of pasture grasses. Stem rust infections can result in seed yield losses of up to 93% in turf-type perennial ryegrass cultivars, forcing seed producers, especially those growing late maturing cultivars, to use fungicides to prevent losses. Incidence of blind seed disease depends on environmental conditions. Infection usually results in seed death from heavily infected stands [44].

Pathogens are not limited to fungi. Bacterial pathogens include *Xanthomonas translucens* pv *graminis*, which causes bacterial wilt disease resulting in yield losses in ryegrass [52]. *Pseudomonas syringae* pv *atropurpurea* causes chlorosis in ryegrass [53]. Viral diseases can also infect perennial grasses, and occur in high incidences causing serious damage. These include barley yellow dwarf virus (BYDV), cereal yellow dwarf virus (CYDV), and ryegrass mosaic virus (RGMV). Upon infection, BYDV and CYDV cause leaf yellowing, stunting and tillering, and these symptoms have an effect on plant performance, productivity, quality and yield [7,54]. Mosaic streaking necrosis by RGMV leads to herbage yield loss [7]. Table 2 further describes common diseases in perennial ryegrass and tall fescue used for pasture and turf, detailing causative organism, symptoms, damage and current control measures.

Phytopathogenic fungi causing disease in grasses may also produce mycotoxins that pose a threat to animal health and wellbeing. *Fusarium* sp. derived toxins—T-2/HT-2, zearalenone, deoxynivalenol—affect food intake and animal performance [55,56]. Though actual toxins are not well characterized, *Drechslera biseptata* has been associated with acute bovine liver disease [57,58]. Rusts caused by *Puccinia* sp., pathogens reduce the palatability of infected grasses by altering total carbohydrate contents [41,59].

Disease outbreaks cause significant yield loss and degrade forage quality, and hence proper control measures are required. The most widely used method for disease control is chemical treatment, which includes spraying fungicides and insecticides for disease vectors [60,61,62]. Fungicides include dimethylation inhibitors, nickel salts and dithiocarbamates [63,64]. Another approach is to apply suitable fertiliser in desirable rates of application to enrich the growth medium with macro and micro nutrients necessary for plant growth and performance [63,65]. Cultural and mechanical control measures include grazing and irrigation management practices [49,60]. All these curative methods are costly and labour demanding. Continuous long term application of fungicides may have a negative impact on livestock health, as some fungicides are toxic when accumulated in large quantities [66,67]. Furthermore, fungicides have a negative impact on *Epichloë* endophytes in perennial ryegrass and tall fescue and may lead to loss of benefits from the symbiotic association [64,68]. Breeding of resistant varieties is time consuming and costly. Thus, introducing beneficial microorganisms as biocontrol agents may be a more cost and time-efficient response.

Bacteria have been reported to be effective biocontrol agents when applied to the plant or seeds. *Pseudomonas aeruginosa* reduces disease incidence and disease severity of grey leaf spot in perennial ryegrass when applied to seeds or the plant in controlled environment pot trials and field trials [69]. *Paenibacillus elgii* SD17 reduces disease severity of brown patch disease and pythium blight in turf grasses in both controlled chamber and field trials [70]. Inoculating novel *Epichloë* strains to grass populations would have relatively minimal ecological impacts as the symbiota are naturally occurring [71].

The asexual *Epichloë* sp. utilized in pastures and turf are known to produce antimicrobial metabolites and inhibit pathogen growth under both in vitro and in planta conditions [25,37,72]. With the availability of new high throughput technology, rigorous analytical methods are available to identify and quantify antimicrobial activity and detect the responsible molecules [73,74,75]. Early studies have shown the potential of *Epichloë* endophytes to inhibit pathogen infections, while more recent studies have focused on isolating and characterising responsible antimicrobial molecules [25,36,72].

## 5. *Epichloë* sp.-Derived Antifungal Activity and Host Disease Resistance Studies

**Figure 1:**
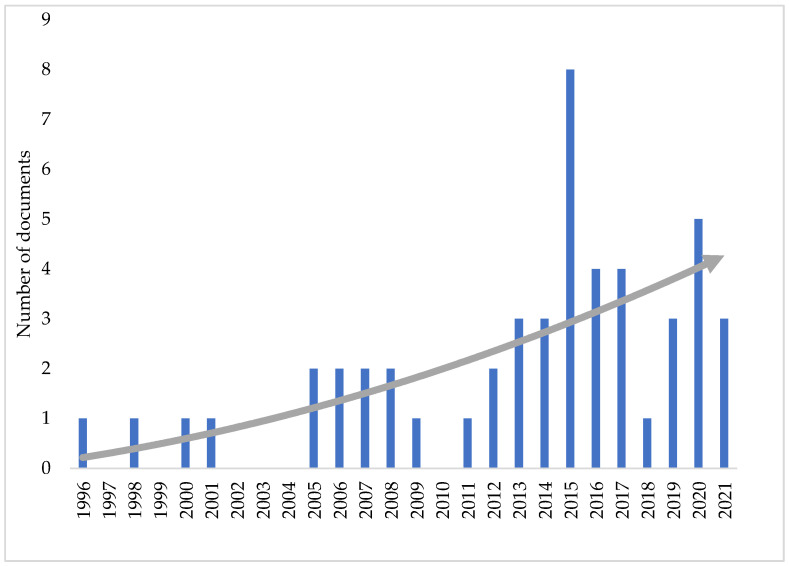
Number of documents (journal articles, letters and short surveys) published in last 25 years on *Epichloë*-derived antifungal activity and/or disease resistance.

There are limited reports of *Epichloë* endophyte-derived antifungal activity and host disease resistance. For this review we used the scientific search engine Scopus (www.scopus.com (accessed on 5 November 2021) to identify peer reviewed scientific reports with the terms “*Epichloë*” or “*Neotyphodium*” and “fungitoxic” or “antifungal” in the title, abstract, or keywords. Only publications in English and published in the last 25 years (1996–2021) were considered. Data integrity and collation were performed by either individually checking and filtering nonrelated articles or preserving and adding related articles cited in reviews and journal publications. Relevant secondary documents listed in Scopus but not indexed in the database were also included. Subsequently, only 46 journal articles, three letters, and one short survey (Supplementary Table S1) were found. During the same time period, the number of English journal manuscripts found using the terms “*Epichloë*” or “*Neotyphodium*” was 1313. Although only 3.5% of *Epichloë* fungi-related studies concerned antifungal activity and host disease resistance against fungal pathogens, this number has been increasing (Figure 1). This demonstrates that researchers are recognizing the importance of *Epichloë* endophyte derived antifungal activity as an important driver for better performing pasture and turf.

## 6. Bioprospecting Antifungal Metabolites from *Epichloë* Endophyte Strains

**Figure 2:**
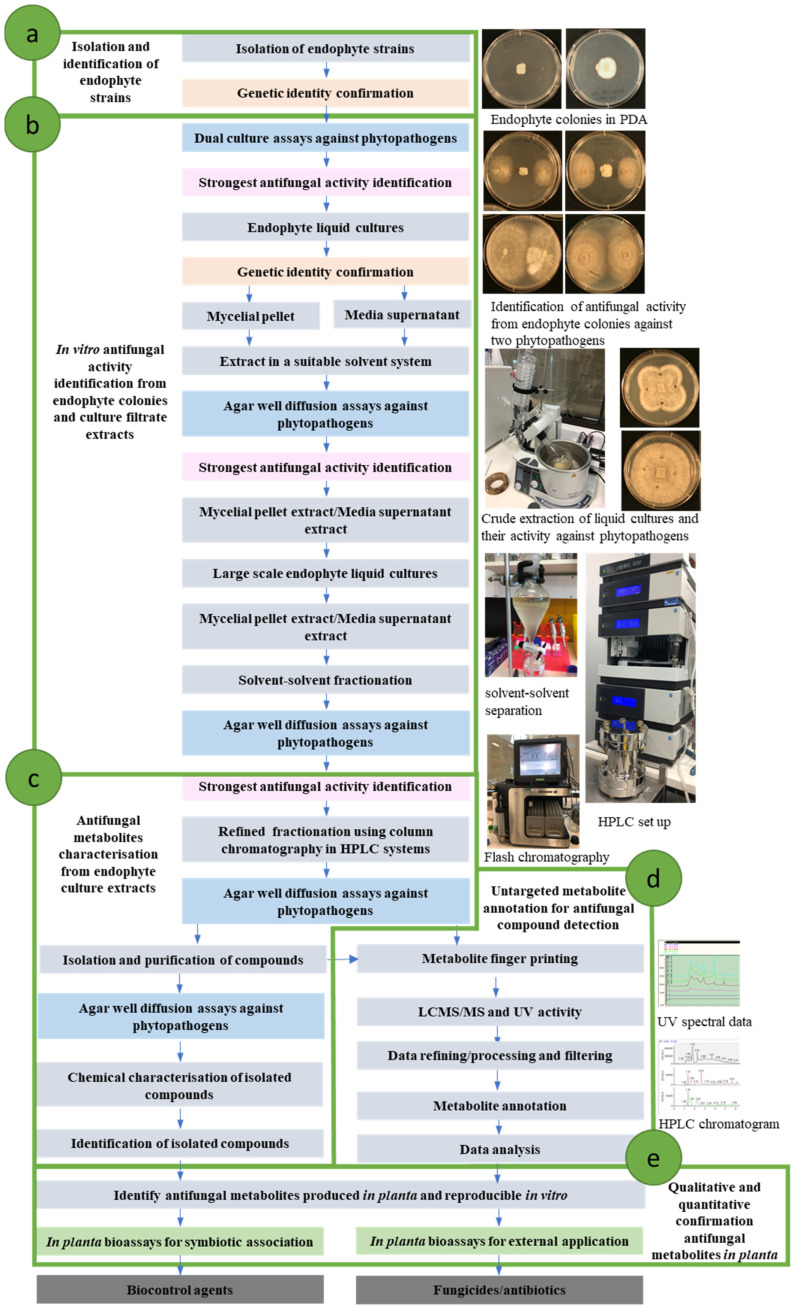
An experimental workflow proposed for bioprospecting antifungal metabolites from *Epichloë* endophytes using a stepwise process. (**a**) *Epichloë* strain identification, (**b**) Identification of bioactive strains using in vitro antifungal activity assays, (**c**) antifungal metabolite isolation and characterisation, (**d**) untargeted metabolite annotation for antifungal compound detection, (**e**) qualitative and quantitative confirmation of antifungal metabolites in planta.

Despite the paucity of publications on *Epichloë*-derived antifungal activity and host disease resistance, there have been papers describing various methodologies to demonstrate the presence of biological activity of *Epichloë* endophyte strains [12,25,91,92,93,94]. However, the methods utilized historically are subjective, qualitative and do not allow for accurate comparisons between studies. To address the current research gaps in *Epichloë*-mediated fungal disease resistance, it is necessary to establish rigorous analytical processes to characterize the bioactivity of *Epichloë* strains quantitatively and accurately to determine the effect of responsible compounds. Recent advances in analytical methods and software tools enable development of standardized protocols and processes to analyze antifungal activity. In this section we review current knowledge in the field, highlighting the methods and tools available, to create a schematic process for identification, characterization and application of antifungal compounds produced by *Epichloë* endophytes (Figure 2).

### 6.1. Epichloë Endophyte Strain Identification

*Epichloë* strains are commonly tested in vitro using different plate-based assays to identify their antifungal activity [92,93,94,95]. Endophyte strains must be isolated, purified, and transferred to in vitro cultures prior to being tested (Figure 2a). As they are naturally found in symbiotic association with host plants, it is ideal to freshly isolate the *Epichloë* strain from an infected plant. [27]. Strain identity should be confirmed in advance of any testing, which generally involves DNA-based PCR analysis, such as an SNP diagnostic test, to enable identification of known *Epichloë* strains [28,96]. Ideally, novel endophyte strains would be defined by whole genome sequencing prior to testing bioactivity. Alkaloid profiling endophyte strains for currently known *Epichloë*-derived alkaloids will provide additional information on animal safety concerns [26,27].

### 6.2. Identification of Bioactive Strains Using In Vitro and in Planta Assays

Antifungal activities of endophyte strains are commonly detected using in vitro dual culture assays [37,92,93,94,95,97]. In dual culture assays, phytopathogens and endophytes are grown in close proximity to study their antagonistic reactions (Figure 2b). Growth parameters (growth area, mycelial density, growth direction) of phytopathogens are observed over a period of time to detect inhibitory activity. Earlier studies qualitatively analysed the results based purely on observation. However, with the availability and accessibility of imaging and image analysis software, growth parameter data such as pathogen growth area can easily be converted to quantitative data. Quantitative data can be analysed for precision and errors, and subjected to statistical analysis to detect the significance of antifungal activity [94]. Dual culture assays are suitable for detecting antifungal activity as they are quick, easily replicated and can be used to test against a range of pathogens. Importantly, the in vitro antifungal phenotypes observed are consistent and observed in independent isolates of the same strain and across duplicate assays [37,98,99].

In addition, dual culture assays provide information on direct interaction of the endophyte with phytopathogens, for example, *Epichloë festucae* strain E437 was shown to reduce hyphal tip growth of the pathogens *Drechslera erythospila* and *Colletotrichum graminicola* [95]. Fernando et al. (2020) observed differential bioactivity between three asexual *Epichloë* strains (Nea 12, Nea 21 and Nea 23) evaluated against three phytopathogens (*Ceratobasidium* sp., *Drechslera* sp. and *Fusarium* sp.), indicating that there is variation in the production of bioactive metabolites and their composition [37]. In vitro liquid culture extracts exhibited differential antifungal activity consistent with dual culture assays [37], establishing that the *Epichloë* strains produce and secrete antifungal metabolites.

However, it should be noted that virulence of a pathogen may be different when it is infecting a plant; therefore, dual culture assay results may not directly relate to pathogen inhibition in planta. Dual culture assays are also unsuitable for biotrophic pathogens (e.g., rusts such as *Puccinia coronata*) that are unable to be grown in pure cultures. Spore germination assays are less common but have also been used to study the antifungal activity of *Epichloë* strains and understand the physiological mechanisms [93,100,101,102]. It is noteworthy that Christensen and Latch’s study in 1991 is the only available in vitro study of *Epichloë coenophiala* (previously known as *Acremonium/Neotyphodium coenophialum*) demonstrating antifungal activity against spores of the rust pathogen *Puccinia graminis* by inhibition of urediniospore germination [101]. Spore germination assays are also a useful tool for testing antifungal compounds isolated from *Epichloë* sp. [102].

Detached leaf assays are another type of semi-in vitro assay used to overcome some of the limitations associated with dual culture and spore germination assays. In detached leaf assays, leaves from endophyte infected host plants are inoculated with phytopathogens and disease symptom development parameters (leaf spot number, area/size of leaf spot) are used to characterise the antifungal activity [25,95]. These assays are complex, do not account for direct interaction of the pathogen with the endophyte [11] and depend on tiller age, endophyte incidence, concentration of pathogen inoculum, and environmental conditions, which may lead to inconsistent results [92]. Nonetheless, detached leaf assays can be informative when performed in addition to dual culture assays.

There are a few instances where pot or field trials were conducted to detect endophyte mediated disease resistance in grasses [91,95,103,104,105,106,107,108,109]. Most studies investigated wild type endophyte strains, and others did not provide information on strain details. These in planta assays have confirmed that *Epichloë* endophytes improve host plant disease resistance. While some studies focused on measuring disease severity parameters (lesion numbers, lesion size) [91,110] other studies included leaf senescence characteristics (chlorophyll content, superoxide dismutase, peroxidase, catalase, and ascorbate peroxidase) to further understand the mechanism of disease resistance [95,103]. Conducting field and pot trials in quarantine is expensive and increases risk of outbreaks.

### 6.3. Antifungal Metabolite Isolation and Characterisation

While most studies have focused on detecting antifungal activity, a few characterised the mode of action and identify compounds responsible. Some studies provide evidence that *Epichloë* strains produce antifungal molecules in vitro liquid cultures and secrete to the culture media [37,97,111,112]. In these studies, the filtrate containing the fungal secretome can be extracted and bioassays performed to identify antifungal activity [37,111,112]. Three studies have extracted the secretome/culture filtrate using different solvent systems and tested their activity against selected grass pathogens [37,112,113]. These confirmed the ability of *Epichloë* strains to produce antifungal molecules in vitro even when not in contact with the pathogens. This characteristic is important to identify and isolate bioactive compounds (Figure 2c).

**Figure 3:**
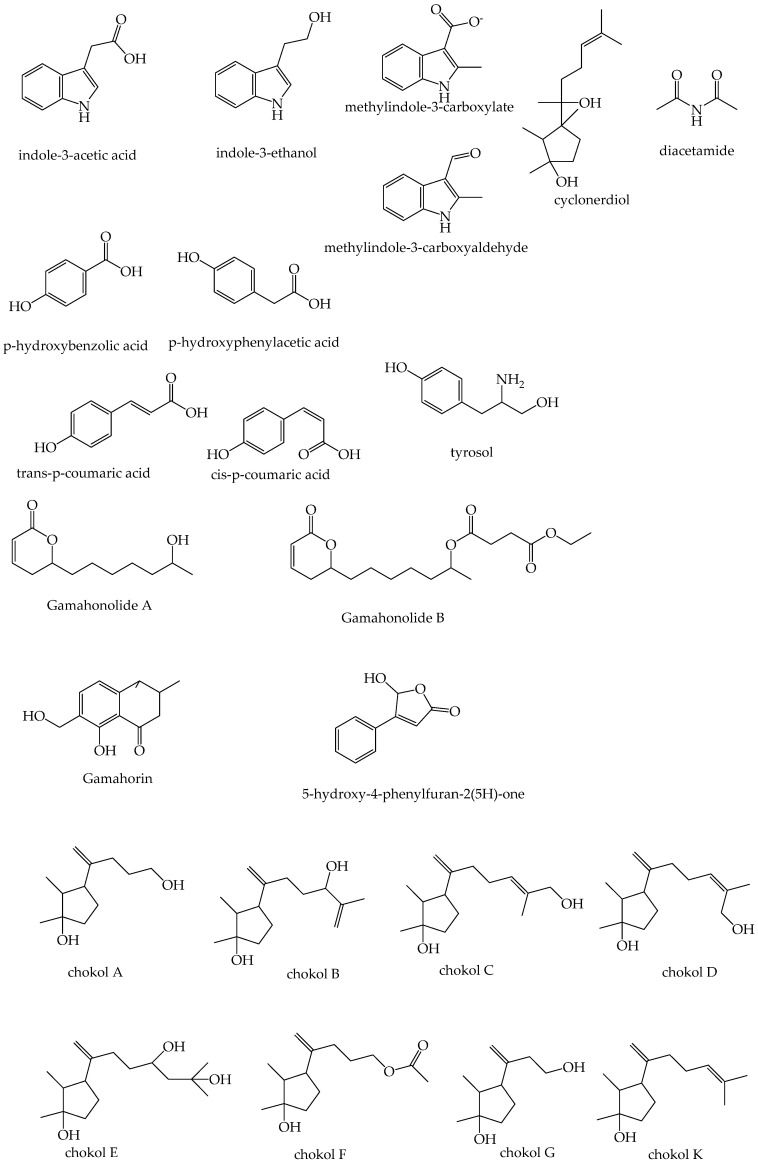
Structures of the fungitoxic compounds isolated from sexual *Epichloë* species that are listed and referenced in Table 3.

Historically, antifungal metabolites have been isolated from a few sexual *Epichloë* strains (Table 3), while antifungal metabolites produced by strains of asexual (endophytic) *Epichloë* species that are utilised by pasture and turf industries remain to be discovered. While most metabolites are isolated from in vitro cultures, others have been isolated from infected plants (Table 3 and Figure 3). Early studies identified a series of antifungal compounds from *Epichloë typhina* isolated from timothy grass (*Phleum pratense*) [114,115]. Yue et al. (2000) tested antifungal activity of three fractions of aqueous extracts from a range of *Epichloë* species and confirmed their antifungal activity against *Cryphonectria parasitica,* the causal phytopathogen of chestnut blight, using thin layer chromatography assay [112]. Subsequently, they used *E. festucae* (BM7, M. D. Richardson) from *Festuca rubra* to isolate six antifungal metabolites and confirmed their activity against *C. parasitica*, *Lactisaria fusiformis*, *Magnaporthe poae*, and *Rhizoctonia solani* using Potato Dextrose Agar (PDA) plate based assays [112]. Nuclear Magnetic Resonance (NMR) and Gas Chromatography-Mass Spectrometry (GC-MS) data were acquired to fully characterise the isolated compounds. Tian et al. (2017) isolated antifungal protein *Efe*-AfpA, active against *Sclerotinia homoeocarpa* [105]. They used *E*. *festucae* Rose City isolate (*E*. *festucae* RC) in association with *Festuca rubra* subsp. *rubra* (strong creeping red fescue) to isolate apoplastic proteins. Sodium dodecyl sulfate-polyacrylamide gel electrophoresis (SDS-PAGE) was used to isolate the proteins. The peptide sequence was determined by using Liquid Chromatography tandem Mass Spectrometry (LC/MS/MS2). Bioactivity of the isolated protein was confirmed using a PDA plate-based assay [105]. Purev et al. (2020) isolated antifungal ε-poly-L-lysines encoded by the *VibA* gene from *E. festucae* strain E437. They used NMR and MALDI-TOF MS to identify the molecule and determine the structure. Disk diffusion assays confirmed the antifungal activity against *Drechslera erythrospila* and *Phytophthora capsica* [116]. Structures of some of these antifungal metabolites are shown in Figure 3. A recent study by Fernando et al. (2021) confirmed currently known *Epichloë*-derived antimammalian and insecticidal alkaloids and their intermediates (peramine, *n*-formylloline, *n*-acetylloline, lolitrem B, epoxyjanthitrem I, paxilline, terpendole E, terpendole C, ergovaline) are not responsible for the antifungal activity observed by *Epichloë* endophytes [22].

### 6.4. Untargeted Metabolite Annotation for Antifungal Compound Detection

*Epichloë* endophytes and their host plants are complex and sophisticated multicellular organisms, thus it is not unexpected that the metabolic profile of endophytes in planta is vastly different and more complex than endophytes in vitro [36,37]. In endophyte-infected plants, both the host plant and endophyte metabolome trigger secondary metabolite biosynthesis machinery that would not be otherwise observed individually [117]. It is important to study the metabolic profiles of these endophyte strains both in vitro, in planta and upon infection with diseases to understand the complex biological process involved in endophyte mediated disease resistance (Figure 2d).

With recent whole-genome sequencing strategies revealing that the number of genes encoding the biosynthetic enzymes in various fungi and bacteria are undoubtedly greater than the known secondary metabolites of these microorganisms, it is highly likely that most endophytes might actually express only a subset of their biosynthetic genes under standard in vitro laboratory conditions, such that only a minor portion of their actual biosynthetic potential is harnessed [118,119]. Thus, selection of the most appropriate method for metabolic profiling and isolation of metabolites, as well as consideration of the environmental conditions the endophytes are grown in, is important to identify the most agronomically important endophytes in the ecosystem.

Metabolomics is the study of metabolite profiles of a cell, tissue or an organism under given conditions [120]. Investigation of metabolic profiles of endophytes in response to a pathogen infection is the first step in understanding the mode of action and enabling their use efficiently against pathogens. Metabolome analysis may entail either a targeted analysis of a certain class of metabolite, or total (untargeted) metabolite profiling of a given sample. Preliminary screening of the total metabolome provides a view on overall performance, whereas targeted bioassay-guided isolations provide more specific details about metabolites and their potential uses [98]. It is important to realise that during the isolation procedure, bioactivity can disappear due to degradation of the bioactive compounds in the extract. Furthermore, the activity can be diluted due to inadequate chromatographic separation and poor fractionation, thus reducing the effective concentration [98].

The most widely used techniques for microbial extracts are High Performance Liquid Chromatography (HPLC), Gas Chromatography (GC), Mass Spectrometry (MS) and Nuclear Magnetic Resonance (NMR) [120]. The more advanced methods couple high resolution mass spectrometers to other analytical techniques, such as chromatography or collision-induced fragmentation, to obtain precision and structural information of compounds in a short period of time with less effort, for example LC-MS, MS2 and GC-MS [120,121]. A qualitative, as well as quantitative, understanding of microbial metabolites requires knowledge of both extracellular and intracellular metabolites [121,122,123]. Hence the microbes, as well as the media they are grown on, are analysed for metabolites. To identify these metabolites, it may be necessary to extract the microbial metabolites into a solvent that is suitable for the specific technique. To extract microbial metabolites, many techniques can be used and there is also a range of possible solvents available depending on the polarity of the metabolites of interest [123].

Pinu et al. describes metabolic footprint analysis as the global identification and quantification of the metabolites present in the spent culture medium of microbial cells using different analytical techniques. Both extracellular as well as intracellular metabolites are specific to a time point under a certain set of environmental conditions while the microbes are growing. Capturing these timepoints is important to conduct a detailed descriptive study; thus, quenching (stopping further biological activity) becomes an important step in microbial metabolite analysis. Depending on the aims, the study could be dynamic (gathering data on microbial growth at multiple time points) or time resolved (on a single time point) [124,125,126]. Selection of an appropriate extraction solvent is important because it affects the yield and polarity of metabolites extracted. Thorough metabolic profiling can unravel the potential of a microorganism (biocontrol agent, natural product uses) and complement genomic studies of the organism.

Untargeted metabolomic study approaches have been used to understand the metabolic potential of *Epichloë* strains relating to antifeeding (insect/invertebrate) activity [127,128,129]. Other studies have investigated the effect of endophytes on the root exudate metabolome [130] and in response to different field nutrient or environmental conditions [128,131]. Untargeted metabolite fingerprinting coupled to spectral data analysis software could be the solution to understanding the complex process of endophyte-host plant-phytopathogen relationships and the metabolic response or production of bioactive metabolites in the presence of a pathogen (Figure 2d). Green et al. (2020) studied the *Lolium perenne* apoplast to identify *Epichloë festucae*-derived novel metabolites [73] and recently Fernando et al. (2021) conducted bioassay guided extraction of antifungal metabolites and metabolite annotation of bioactive extracts from *Epichloë* strains [36].

The analytical tools used to detect metabolites in these studies include ultra-high performance liquid chromatography systems (UHPLC), photodiode array detector, Q Exactive Plus high-resolution mass spectrometer (QE-MS) and high-resolution orthogonal time-of-flight MS (e.g., HRqTOF-MS). There are many data mining software platforms available to obtain a list of metabolites or features including vendor specific software such as MARKERLYNX XS for MASSLYNX v.4.1. (Waters, MA, USA), or vendor neutral software such as Refiner MS and Analyst modules of Genedata Expressionist^®^ (Genedata, Basel, Switzerland). Metabolites can be annotated based on accurate mass information and their identity defined by database searches. Green et al. (2020) used databases (KEGG, BioCyc and in-house databases) (http://www.kegg.jp) (http://biocyc.org) to annotate metabolites and identified a novel amino acid glycoside, a set of *Epichloë* cyclins, peramine, and putatively identified two peptides, a Kojibiose-related metabolite (322.0643 Da), N-(hydroxypentyl) acetamide (145.1101 Da), and a compound with an accurate mass or m/z of 471.1952 Da [73]. Fernando et al. annotated metabolites from two bioactive strains of asexual *Epichloë* species and used them as biomarkers for detection in planta. Bioassay guided fractionation, followed by metabolite analysis, identified 61 “known unknown” prospective antifungal metabolites (out of more than 20,000 in planta) that, either singly or in combination, are responsible for the observed bioactivity. The workflow developed in this study allows testing of endophyte bioactivity while ensuring that the metabolite is expressed in planta and so useful for field deployment (Figure 2).

These studies show how metabolite fingerprinting can be used for bioprospecting antifungal metabolites from *Epichloë* endophytes. While isolation and full chemical characterisation is still necessary to understand the role and mode of action of the bioactive metabolites, these robust novel methods help visualise the potential of novel *Epichloë* strains as biocontrol agents without conducting field or pot trials. Based on this, we propose a methodology of exhaustive testing and analysis to identify biologically active compounds (Figure 2).

### 6.5. Qualitative and Quantitative Confirmation of Antifungal Metabolites in Planta

It is a common practice to conduct routine alkaloid testing for the ‘known known’ *Epichloë*-derived toxic alkaloids before field deployment of novel associations [26,27,34]. Comprehensively characterized and purified compounds can be applied in routine diagnostics for the presence and abundance of *Epichloë*-derived antifungal metabolites in planta (Figure 2e) [36]. Availability of well tested antifungal compound standards, or availability of isolation methods through scientific publication, enables further investigation of abundance of antifungal compounds in response to environmental conditions, such as disease challenge by phytopathogens, and will confirm their role in improving host plant disease resistance.

## 7. Future Directions

In the past 25 years, the potential of asexual *Epichloë* sp. to provide bioprotection from fungal pathogens to the host grass has been noted frequently but not pursued. Wildtype strains have been shown to improve disease control in pot trials and field scenarios, often when outbreaks have occurred rather than by design. There is a strong market globally for novel, animal safe, endophyte strains that provide insect and disease control. Some of these strains exhibit strong antifungal activity against phytopathogens, as observed using in vitro bioassays. With the discovery of novel endophytes with favourable ‘known known’ alkaloid profiles, and significant advances in high-throughput analytical techniques and data analysis, the opportunity now arises to investigate endophyte-mediated disease resistance. This knowledge can then be applied to select superior strains for the pasture and turf industries, improving animal health and host grass performance through enhanced disease resistance. It is anticipated that the outcomes of these studies would expand the current screening methods for *Epichloë* sp. strains to include bioprotective compounds and their respective genes.

## Figures and Tables

**Table 1:** Novel *Epichloë* endophyte strains for which Plant Breeders’ Rights (PBR) have been granted worldwide ^1^.

Country	PBR Grant Date	Endophyte Strain (Market Name)	*Epichloë* Species	Host Common Name	Known Alkaloid Profile ^2^	Applicant
New Zealand	23 April 1996 (expired)	AR1	*E. festucae* var. *lolii* (*Lp*TG-1)	Perennial ryegrass	P	Grasslanz Technology Ltd. (GTL; Palmerston North, New Zealand)
European Union	20 October 2003					
Australia	26 October 2004					
New Zealand	21 April 2015	AR1006	*E. uncinata*	Meadow fescue	L	GTL
New Zealand	5 October 2016	AR1017	*E. uncinata*	Meadow fescue	L	GTL
New Zealand	29 November 2018	AR127	*E. festucae* var. *lolii* (*Lp*TG-1)	Perennial ryegrass		GTL
New Zealand	25 July 2008	AR37	*E. festucae* var. *lolii* (*Lp*TG-1)	Perennial ryegrass	J	GTL
Australia	30 March 2010					
Uruguay	20 November 2011					Fischer Fleurquin Gustavo (FFG; Montevideo, Uruguay)
New Zealand	23 April 1996 (expired)	AR501	*E. coenophiala* (*Fa*TG-1)	Tall fescue	LP	GTL
European Union	20 October 2003					
New Zealand	1 February 1999 (expired)	AR542 (MaxP^®^ New Zealand, Australia; MaxQ^®^ USA)	*E. coenophiala* (*Fa*TG-1)	Tall fescue	LP	GTL
Australia	26 October 2004					
Uruguay	6 December 2004					FFG
New Zealand	25 July 2008	AR584 (MaxP^®^ New Zealand, Australia; MaxQII™ USA)	*E. coenophiala* (*Fa*TG-1)	Tall fescue	LP	GTL
Australia	29 September 2010					
Uruguay	2 August 2013					FFG
Argentina	4 September 2014					Gentos SA (Buenos Aires, Argentina)
New Zealand	12 May 2010	AR601 (Avanex^®^)	*E. coenophiala* (*Fa*TG-1)	Tall fescue	EL	GTL
Australia	19 August 2013					
European Union	22 May 2017					
New Zealand	12 May 2010	AR604	*E. coenophiala* (*Fa*TG-1)	Tall fescue	EL	GTL
European Union	22 May 2017					
New Zealand	28 August 2014	AR95 (Avanex^®^)	*E. festucae* var. *lolii* (*Lp*TG-1)	Perennial ryegrass	E	GTL
European Union	22 May 2017					
Australia	3 April 2018					
Uruguay	21 November 2014					FFG
New Zealand	17 January 2019	CM142	*E. festucae* var. *lolii* (*Lp*TG-1)	Perennial ryegrass	J	Cropmark Seeds Ltd. (CSL; Rolleston, New Zealand)
Australia	17 August 2020					Cropmark Seeds Australia Pty Ltd. (CSA; South Melbourne, Australia)
New Zealand	27 August 2014	E815 (Edge)	*E. festucae* var. *lolii* (*Lp*TG-1)	Perennial ryegrass	LtmEP	DLF Seeds A/S (DLF; Roskilde, Denmark)
Australia	23 October 2017					DLF
New Zealand	23 June 2010	Happe	*E. siegelii*		L	DLF
Australia	23 October 2017					DLF
New Zealand	25 July 2008	Nea 2 (NEA/NEA2/NEA4) ^3^	*E. festucae* var. *lolii* (*Lp*TG-1)	Perennial ryegrass	LtmEP	Barenbrug New Zealand Ltd. (BBNZ; Christchurch, New Zealand)
New Zealand	30 June 2009	Nea 3 (NEA4 ^3^)	*E. festucae* var. *lolii* (*Lp*TG-1)	Perennial ryegrass	EP	BBNZ
New Zealand	25 July 2008	Nea 6 (NEA2 ^3^)	*E. festucae* var. *lolii* (*Lp*TG-1)	Perennial ryegrass	EP	BBNZ
New Zealand	29 August 2014	Nea 10	*E. festucae* var. *lolii* (*Lp*TG-1)	Perennial ryegrass	EP	BBNZ
New Zealand	13 August 2014	Nea 11	*E. festucae* var. *lolii* (*Lp*TG-1)	Perennial ryegrass	EP	BBNZ
New Zealand	19 March 2021	Nea 12	*Epichloë* sp. (*Lp*TG-3)	Perennial ryegrass	J	Agriculture Victoria Services Pty Ltd. (Bundoora, Australia)
New Zealand	29 August 2014	Nea 21	*Epichloë* sp. (*Fa*TG-3)	Tall fescue	LP	BBNZ
New Zealand	29 August 2014	Nea 23	*Epichloë* sp. (*Fa*TG-3)	Tall fescue	LP	BBNZ
New Zealand	10 July 2019	Nea 47 (NEA2 ^3^)	*E. festucae* var. *lolii* (*Lp*TG-1)	Perennial ryegrass	EP	BBNZ
New Zealand	18 August 2014	PTK647 (Protek^®^)	*E. coenophiala* (*Fa*TG-1)	Tall fescue	EL	DLF
Australia	23 October 2017					DLF
New Zealand	28 August 2014	U12	*E. uncinata*	Meadow fescue	L	CSL
Australia	12 August 2021					CSA
New Zealand	2 September 2016	U13	*E. uncinata*	Meadow fescue	L	CSL
New Zealand	25 July 2008	U2 (GrubOUT^®^)	*E. uncinata*	Meadow fescue	L	CSL
Australia	30 January 2014					CSA
Argentina	11 March 2015					Gentos SA
European Union	22 May 2017					CSL
New Zealand	14 October 2008	UNC1	*E. uncinata*	Meadow fescue	L	CSL
Australia	Not applicable ^4^	AR5 (Endo5)	*E. festucae* var. *lolii* (*Lp*TG-1)	Perennial ryegrass	EP	GTL
USA	Not applicable ^4^	E34^®^	*E. coenophiala* (*Fa*TG-1)	Tall fescue	LP	Barenbrug USA (Tangent, OR, USA)
USA	Not applicable ^5^	KY31 (Kentucky31)	*E. coenophiala* (*Fa*TG-1)	Tall fescue	EPL	Not applicable
worldwide	Not applicable ^5^	SE (Standard endophyte)	*E. festucae* var. *lolii* (*Lp*TG-1)	Perennial ryegrass	LtmEP	Not applicable

^1^ UPOV Pluto database search for Plant Breeders’ Rights granted to 26 November 2021 https://pluto.upov.int/search (Search: Botanical name -*Epichloë*). ^2^ Alkaloid profile, P = peramine, L = lolines, E = ergovaline, Ltm = lolitrem B, J = epoxy-janthitrems. ^3^ Strains are sometimes marketed as a combination e.g., NEA2 = Nea 2, Nea 6, Nea 47. ^4^ Commercial endophytes that were not listed in UPOV Pluto database search results. ^5^ Wildtype, toxic strains.

**Table 2:** Common disease-causing phytopathogens in pasture and turf grasses.

Disease/ Common Name	Causative Organism	Symptoms	Damage/Loss	Control Measures	References
Crown rust	*Puccinia coronata*	Reddish brown spores on leaf	Dry matter yield loss 30–40% and stock thrift	Fungicide; Judicious grazing management; Resistant varieties.	[41,45,76]
Grey leaf spot	*Pyricularia grisea*	Small water-soaked lesions on leaf blades gradually turning to dark necrotic spots and to grey spots	Up to 90% pasture loss	Fungicide; Controlled release of N fertiliser; Biocontrol, Resistant varieties.	[50,63,77,78]
Brown blight and net blotch	*Drechslera* sp.	Net lesions with small dark brown bars amphigenous lesions with dark brown margins, light brown center	Dry matter and herbage loss	Fungicide; Managed grazing before it spreads.	[49,60,79]
Stem end rust	*Puccinia graminis*	Reddish brown spores on sheath and stem	Seed yield loss, Dry matter loss	Fungicide; Judicious grazing management; Resistant varieties.	[49,80]
Blind seed disease	*Gloeotinia temulenta*	Fungal mycelia on seeds under microscopic observation	Seed yield loss, reduce seed germination 50–90%	Fungicide; Increased rate of N application.	[64,65,81]
Snow mould	*Microdochium nivale*	Dark brown lesions and pink sporodochia rows parallel to veins	Seedling damage leading to yield loss, seed loss and yield loss	Fungicide; Biological control; Compost application.	[51,60,82]
Yellow patch	*Ceratobasidium cereale*	Root pathogen	Yield loss	Fungicide	[83,84]
seedling pathogen and leaf spot	*Fusarium solani*	Wilting of seedlings and necrotic lesions in mature plants	Yield loss, dry matter loss	Fungicide	[85,86]
Bacterial wilt	*Xanthomonas translucens*	Water-soaked lesions and turning to bluish purple colour	Forage yield loss 20–40%	Biological control; Resistant varieties.	[52,87,88,89]
Ryegrass mosaic virus	RGMV	Light green-yellow streaky mosaic or brown necrosis on leaves	Dry matter yield loss 21–30%	Resistant varieties; Mixed pasture species.	[7,90]

**Table 3:** Summary of fungitoxic compounds isolated from sexual *Epichloë* sp. and their characteristics.

Fungitoxic Compound	Chemical Characteristics	Chemical Formula	*m*/*z* or Molecular Weight	*Epichloë* sp.	Host Grass	Source Material	Tested Pathogens	Reference
Indole-3-acetic acid	IAA derivative	C_10_H_9_NO_2_	176.0667	*E. festucae*	*Festuca rubra*	purified mycelia	*Lactisaria fusiformis*, *Magnaporthe poae*, *Rhizoctonia solani*	[112]
Indole-3-ethanol	IAA derivative	C_10_H_11_NO	162.0874	*E. festucae*	*Festuca rubra*	purified mycelia	*L. fusiformis*, *M. poae*, *R. solani*	[112]
Methylindole-3-carboxylate	IAA derivative	C_10_H_9_NO_2_	175.0594	*E. festucae*	*Festuca rubra*	purified mycelia	*L. fusiformis*, *M. poae*, *R. solani*	[112]
Indole-3-carboxaldehyde	IAA derivative	C_9_H_7_NO	159.0684	*E. festucae*	*Festuca rubra*	purified mycelia	*L. fusiformis*, *M. poae*, *R. solani*	[112]
Cyclonerodiol	sesquiterpinoid	C_15_H_28_O_2_	165.0507	*E. festucae*	*Festuca rubra*	purified mycelia	*L. fusiformis*, *M. poae*, *R. solani*	[112]
Chokol A Chokol B Chokol C Chokol D Chokol E Chokol F Chokol G	sesquiterpinoid	C_12_H_22_O_2_ C_15_H_26_O_2_ C_15_H_26_O_2_ C_15_H_26_O_2_ C_15_H_28_O_3_ C_14_H_24_O_3_ C_11_H_20_O_2_	199.1693 239.2006 239.2006 239.2006 271.2228 241.1798 185.1536	*E. typhina*	*Phleum pratense*	*Epichloë* infected plant material (choke)	*Cladosporium herbarum, Cladosporium phlei*	[114,132]
Chokol K	sesquiterpinoid	C_15_H_26_O	222.1984 ^1^ [M-H_2_O]^−^ = 204	*E. sylvatica* *E. clarkii*	*Brachypodium sylvaticum* *Holcus lanatus*	Unfertilized stromata and unfertilized stromata head space	*Stagonospora nodorum*, *Mycosphaerella graminicola*	[102]
*N,N*-diacetamide	diactamide	C_4_H_7_NO_2_	102.0610	*E. festucae*	*Festuca rubra*	*Epichloë* infected plant material (leaves)	*L. fusiformis*, *M. poae*, *R. solani*	[112]
Gamahonolide A Gamahonolide B Gamahorin	gamahonolide	C_12_H_2 3_ C_18_H_28_O_6_ C_12_H_14_O_4_	213.1502 341.1952 222.0881	*E. typhina*	*Phleum pratense*	*Epichloë* infected plant material (choke)		[133]
5-hydroxy-4-phenyl-2(5H)-furanone		C_12_H_10_O_3_	177.0546	*E. typhina*	*Phleum pratense*	*Epichloë* infected plant material (choke)		[133]
Trans-p-coumaric acid Cis-p-coumaric acid p-hydroxybenzoic acid p-hydroxyphenylacetic acid Tyrosol	phenolic acid derivatives	C_9_H_8_O_3_ C_9_H_8_O_3_ C_7_H_6_O_3_ C_8_H_8_O_3_ C_8_H_10_O_2_	164.1580 164.1580 139.1220 153.0507 168.0980	*E. typhina*	*Phleum pratense*	*Epichloë* infected plant material (choke)	*C. phlei*	[134]
Epichlicin	Cyclic peptide	C_48_H_74_N_12_O_14_	^3^ [M+Na+17]^+^ = 1082	*E. typhina*	*Phleum pretense*	purified mycelia	*C. phlei*	[135]
Fatty acids	C-18 and C-19 fatty acid	C_18_H_32_O_3_ C_19_H_34_O_3_ C_l9_H_36_O_3_ C_l9_H_36_O_3_	297 311 312 312	*E. typhina*	*Phleum pratense*	*Epichloë* infected plant material (choke)	*C. herbarum, C. phlei*	[115]
Efe-AfpA	protein	55 amino acids	6278 Da	*E. festucae*	*Festuca rubra* subsp. *rubra*	*Epichloë* infected plant material (tiller)	*Sclerotinia homoeocarpa*	[105,136]
Cyclosporin T	peptides	C_61_H_109_N_11_O_12_ (11 amino acids)	1188.6 g/mol (^2^ MW)	*E. bromicola*	*Elymus tangutorum*	purified mycelia	*Alternaria alternata*, *Bipolaris sorokiniana*, *Fusarium avenaceum* *Curvularia lunata*	[136]
ε-poly-L-lysines	peptides	28–34 lysine sub-units		*E. festucae*	*Festuca pulchella*	purified mycelia	*Drechslera erythrospila* *Phytophthora capsici*	[116]

^1^*m*/*z* when ionised to [M-H_2_O]^−^, ^2^ Molecular weight indicated in g/mol, ^3^ *m*/*z* when ionised to [M+Na+17]^+^.

## Data Availability

Data presented in this review is contained within the article and supplementary material.

## Supplementary Materials

The following supporting information can be downloaded at: https://www.mdpi.com/article/10.3390/microorganisms10010064/s1, Supplementary Table S1: List of peer reviewed scientific reports in the last 25 years (1996–2021) with the terms “*Epichloë*” OR “*Neotyphodium*” AND “fungitoxic” OR “antifungal” in the title, abstract, or keywords.
