# Supplementary material for: Metabolic Potential of Epichloë Endophytes for Host Grass Fungal Disease Resistance
Source: Microorganisms. 2021 Dec 29;10(1):64. doi: 10.3390/microorganisms10010064 (PMC8781568; doi:10.3390/microorganisms10010064)
Supplement: Supplementary file 1 [file microorganisms-10-00064-s001.zip › microorganisms-1509766-supplementary.pdf]

Supplementary Materials: Metabolic potential of *Epichloë* endophytes for host grass disease resistance

Krishni Fernando, Priyanka Reddy, Simone J. Rochfort, German C. Spangenberg and Kathryn M. Guthridge

Supplementary Table S1: List of peer reviewed scientific reports in the last 25 years (1996 – 2021) with the terms "*Epichloë*" OR "*Neotyphodium*" AND "fungitoxic" OR "antifungal" in the title, abstract, or keywords

First author name	Year	Title	Source title	Cited	Source
Wang, Y.	2021	Preliminary evaluation of the disease resistance of *Festuca sinensis* infected by *Epichloë sinensis*	Journal of Phytopathology	1	Scopus
Kou, M. Z.	2021	The plant salicylic acid signalling pathway regulates the infection of a biotrophic pathogen in grasses associated with an *Epichloë* endophyte	Journal of Fungi	2	Scopus
Wang, R.	2021	The *Epichloë festucae* antifungal protein efe-AfpA is also a possible effector protein required for the interaction of the fungus with its host grass *Festuca rubra* subsp. *rubra*	Microorganisms	1	Scopus
Li, F.	2020	Effects of the fungal endophyte *Epichloë festucae* var. *lolii* on growth and physiological responses of perennial ryegrass cv. fairway to combined drought and pathogen stresses	Microorganisms	2	Scopus
Shi, X.	2020	Endophytic fungi activated similar defense strategies of *Achnatherum sibiricum* host to different trophic types of pathogens	Frontiers in Microbiology	3	Scopus
Fernando, K.	2020	Novel antifungal activity of *Lolium*-associated *Epichloë* endophytes	Microorganisms	4	Scopus
Purev, E.	2020	Identification of ε-Poly-L-lysine as an antimicrobial product from an *Epichloë* endophyte and isolation of fungal ε-PL synthetase gene	Molecules	8	Scopus
Song Q. Y.	2020	Do *Epichloë* endophytes and their grass symbiosis only produce toxic alkaloids to insects and livestock?	Journal of Agricultural and Food Chemistry	4	Scopus
Guo, Y.	2019	Effects of AM fungi and grass endophytes on perennial ryegrass *Bipolaris sorokiniana* leaf spot disease under limited soil nutrients	European Journal of Plant Pathology	5	Scopus
Twarużek, M.	2019	Evaluation of cytotoxicity and mould contamination of selected plants from meadows covered by the agri-environmental program	Toxins	1	Scopus
Qin, J.	2019	Endophyte infection and methyl jasmonate treatment increased the resistance of *Achnatherum sibiricum* to insect herbivores independently	Toxins	9	Scopus
Kauppinen, M.	2018	*Epichloë* endophyte effects on leaf blotch pathogen (*Rhynchosporium* sp.) of tall fescue (*Schedonorus phoenix*) vary among grass origin and environmental conditions	Plant Ecology and Diversity	4	Scopus
Chen, W.	2017	The advantages of endophyte-infected over uninfected tall fescue in the growth and pathogen resistance are counteracted by elevated CO_2	Scientific Reports	9	Scopus
Tian, Z.	2017	The *Epichloë festucae* antifungal protein has activity against the plant pathogen *Sclerotinia homoeocarpa*, the causal agent of dollar spot disease	Scientific Reports	20	Scopus
Iannone, L.J.	2017	Seed-transmitted *Epichloë* sp. endophyte alleviates the negative effects of head smut of grasses (*Ustilago bullata*) on *Bromus auleticus*	Fungal Ecology	9	Scopus

Perez, L.I.	2017	Symbiosis with systemic fungal endophytes promotes host escape from vector-borne disease	Oecologia	8	Scopus
Xia, C.	2016	An *Epichloë* endophyte improves photosynthetic ability and dry matter production of its host *Achnatherum inebrians* infected by *Blumeria graminis* under various soil water conditions	Fungal Ecology	41	Scopus
Wang, X.	2016	Pathogen resistant advantage of endophyte-infected over endophyte-free *Leymus chinensis* is strengthened by pre-drought treatment	European Journal of Plant Pathology	23	Scopus
Lugtenberg, B.J.J.	2016	Fungal endophytes for sustainable crop production	FEMS Microbiology Ecology	111	Scopus
Pérez, L. I.	2016	Can the defensive mutualism between grasses and fungal endophytes protect non-symbiotic neighbours from soil pathogens?	Plant and Soil	4	Review/google scholar
Song, Q. Y.	2015	Antifungal, phytotoxic, and cytotoxic activities of metabolites from *Epichloë bromicola*, a fungus obtained from *Elymus tangutorum* grass	Journal of Agricultural and Food Chemistry	29	Scopus
Zhou, L.Y.	2015	Antifungal activity and phytochemical investigation of the asexual endophyte of *Epichloë* sp. from *Festuca sinensis*	Science China Life Sciences	18	Scopus
Zhang, X.X.	2015	Chemical composition and antifungal activity of the volatile oil from *Epichloë gansuensis*, endophyte-infected and non-infected *Achnatherum inebrians*	Science China Life Sciences	6	Scopus
Ma, M.	2015	Effects of the endophyte *Epichloë festucae* var. *lolii* of perennial ryegrass (*Lolium perenne*) on indicators of oxidative stress from pathogenic fungi during seed germination and seedling growth	European Journal of Plant Pathology	36	Scopus
Xia, C.	2015	*Epichloë* endophyte affects the ability of powdery mildew (*Blumeria graminis*) to colonise drunken horse grass (*Achnatherum inebrians*)	Fungal Ecology	37	Scopus
Wiewióra, B.	2015	Endophyte-mediated disease resistance in wild populations of perennial ryegrass (*Lolium perenne*)	Fungal Ecology	34	Scopus
Niones J.T.	2015	VibA, a homologue of a transcription factor for fungal heterokaryon incompatibility, is involved in antifungal compound production in the plant-symbiotic fungus *Epichloë festucae*	Eukaryotic Cell	12	Scopus
Zhang, X. X.	2015	Effects of symbiotic *Epichloë gansuensis* endophyte on drunken horse grass (*Achnatherum inebrians*) growth and seed production	New Zealand Journal of Agricultural Research	8	Review/google scholar
Zhang, X.X.	2014	Antifungal activity of petroleum ether extracts from *Achnatherum inebrians* infected with *Neotyphodium gansuense*	Science China Life Sciences	4	Scopus
Tanentzap, A.J.	2014	Ungulate saliva inhibits a grass-endophyte mutualism	Biology Letters	6	Scopus
Niones, J.T.	2014	An isolate of *Epichloë festucae*, an endophytic fungus of temperate grasses, has growth inhibitory activity against selected grass pathogens	Journal of General Plant Pathology	23	Scopus
Pańka, D.	2013	Occurrence of *Neotyphodium* and *Epichloë* fungi in meadow fescue and red fescue in poland and screening of endophyte isolates as potential biological control agents	Acta Scientiarum Polonorum, Hortorum Cultus	7	Scopus
Pańka, D.	2013	Susceptibility of tall fescue to *Rhizoctonia zeae* infection as affected by endophyte symbiosis	Annals of Applied Biology	20	Scopus
Pańka, D.	2013	Production of phenolics and the emission of volatile organic compounds by perennial ryegrass (*Lolium perenne* L.)/*Neotyphodium lolii* association as a response to infection by *Fusarium poae*	Journal of Plant Physiology	61	Scopus

Sabzalian, M. R.	2012	Reaction to powdery mildew fungus, *Blumeria graminis* in endophyte-infected and endophyte-free tall and meadow fescues	Australasian Plant Pathology	5	Review/google scholar
Deshmukh, S.K.	2012	Fungal endophytes: A potential source of antifungal compounds	Frontiers in Bioscience - Elite	14	Scopus
Pańka, D.	2011	Effect of *Neotyphodium uncinatum* endophyte on meadow fescue yielding, health status and ergovaline production in host-plants	Journal of Plant Protection Research	14	Review/google scholar
Ren, An-Zhi	2009	Difference in antifungal activity of morphotypes of clavicipitaceous endophytes within and between species	Acta Ecologica Sinica	1	Review/google scholar
Tian, P.	2008	Effect of the endophyte *Neotyphodium lolii* on susceptibility and host physiological response of perennial ryegrass to fungal pathogens	European Journal of Plant Pathology	51	Scopus
Steinebrunner, F.	2008	Ecological role of volatiles produced by *Epichloë*: Differences in antifungal toxicity	FEMS Microbiology Ecology	27	Scopus
Li, C.J.	2007	Interactions of *Neotyphodium gansuense*, *Achnatherum inebrians*, and plant-pathogenic fungi	Mycological Research	45	Scopus
Seto, Y.	2007	Novel cyclic peptide, epichlicin, from the endophytic fungus, *Epichloë typhina*	Bioscience, Biotechnology and Biochemistry	34	Scopus
Clarke, B.B.	2006	Endophyte-mediated suppression of dollar spot disease in fine fescues	Plant Disease	111	Scopus
Wäli, P.R.	2006	Susceptibility of endophyte-infected grasses to winter pathogens (snow molds)	Canadian Journal of Botany	55	Scopus
Bonos, S. A.	2005	Suppression of red thread in fine fescues through endophyte-mediated resistance	Applied Turfgrass Science	54	Review/google scholar
Seto, Y.	2005	Production of phleichrome by *Cladosporium phlei* as stimulated by diketopiperadines of *Epichloë typhina*	Bioscience, Biotechnology and Biochemistry	14	Scopus
Hiatt, E.E.	2001	Monoclonal antibodies incorporated into *Neotyphodium coenophialum* fungal cultures: Inhibition of fungal growth and stability of antibodies	Fungal Genetics and Biology	11	Scopus
Yue, Q.	2000	Isolation and characterization of fungal inhibitors from *Epichloë festucae*	Journal of Agricultural and Food Chemistry	118	Scopus
Kanada, R.M.	1998	Asymmetric hydrogen transfer protocol for enantiocontrolled synthesis of chokol G	Chemical Communications	23	Scopus
Christensen M.J.	1996	Antifungal activity in grasses infected with *Acremonium* and *Epichloë* endophytes	Australasian Plant Pathology	55	Scopus
